# The triceps reflecting approach (Bryan-Morrey) for distal humerus fracture osteosynthesis

**DOI:** 10.1186/1471-2474-15-406

**Published:** 2014-12-04

**Authors:** Lukas Daniel Iselin, Tobias Mett, Reto Babst, Marcel Jakob, Daniel Rikli

**Affiliations:** Department of Traumatology, University Hospital Basel, Spitalstrasse 21, CH-3041 Basel, Switzerland; Department of Traumatology, Luzerner Kantonsspital Luzern, Luzern, CH-6000 Switzerland

**Keywords:** Bryan-Morrey, Triceps sparing, Distal humerus, Fracture, Morbidity

## Abstract

**Background:**

“Chevron”-Olecranon osteotomies are commonly used for the approach to intraarticular distal humerus fractures but are often associated with procedure related complications. We studied the triceps reflecting approach (TRA) with preservation of the extensor apparatus as a safe alternative giving a sufficient exposure to the elbow joint and helping to achieve anatomical fracture reduction with the intact olecranon as a template.

**Methods:**

We performed a retrospective review at two trauma centres and identified 31 skeletally mature distal humerus fractures treated with a TRA. 24 of the patients returned to follow-up including history recording, physical examination with functional analysis of the operated vs the normal site with the DASH and Mayo scores, measurement of range of motion, isometric elbow strength measurement and radiographic documentation.

**Results:**

Mean duration of FU was 51 months (24 months-12 years) in 24 patients, 13 female, 11 male with an average age of 57.7 years (range 17-89). AO Classification showed five A2, one B2, two C1, 9 C2 and 7 C3 fractures. Radiologic control showed adequate reduction, distal humeral alignment and fracture healing in all patients. The strength analysis of flexion and extension revealed no statistically relevant loss of strength at last FU. Range of motion was excellent.

**Conclusion:**

The TRA is a valuable option for ORIF in distal intraarticular humerus fractures. It preserves the normal joint anatomy of the olecranon and avoids the potential complications associated with olecranon osteotomy. The clinical outcome parameters of our series revealed excellent maintenance of strength compared to the contralateral side.

## Background

“Chevron”-olecranon osteotomies are commonly used for the approach to intraarticular distal humerus fractures but they are often associated with procedure related complications or complaints [1–3]. Current concepts of distal humerus fracture treatment dated from 2011 see fair evidence to suggest that the use of a triceps-splitting approach leads to functional outcomes similar to those provided by olecranon osteotomy while potentially avoiding the complications associated with the “Chevron”-olecranon osteotomy. The use of a triceps-splitting approach might lead to functional outcomes that are better than those following an olecranon osteotomy in the treatment of distal humeral fractures [4]. In a posttraumatic setting or in osteoarthritis triceps sparing approaches for elbow arthroplasty are commonly used with good to excellent results [5–7].

Until to date only limited data have been available on triceps sparing approaches for adult distal humerus fracture treatment.

The aim of our study was to answer the following main questions

1. does the triceps reflecting approach offer sufficient exposure of the joint surface for anatomic reduction and stable internal fixation of the fracture; and,
2. does the triceps reflecting approach lead to functional impairment of the extensor apparatus as measured objectively.and to assess if this approach might reduce the complication rate compared to the known data from current literature [8].

## Methods

This bi-center study was performed as a retrospective review at two level-1 trauma centres from 2002 to 2012. We identified 31 skeletally mature consecutive patients with distal humerus fractures treated with a triceps reflecting approach. In all participants informed consent for participation in the study was obtained. The study was performend in compliance with the Helsinki Declaration and local ethical committee approval (Ethical Committee Northern Switzerland, EKNZ 035-2014) to access our hospital’s records for this retrospective study.

Inclusion criteria: All consecutive patients at the 2 centers from 2002 on till 2012 with ORIF and a triceps reflecting approach are included in the study and analysed. Exclusion criteria: Open fractures and additional injuries of the upper extremities were excluded from the analysis. Patient records and radiographs were reviewed to determine injury and operative characteristics, complications, and adequacy of articular reduction. Twenty-four patients returned to follow-up. Four patients passed away unrelated to the trauma, two were mentally impaired to such an extent that a precise examination could not be performed. One patient could not to be retrieved by the investigators. The follow up included history recording, physical examination of the operated vs the not harmed opposite side including strengths measurement and ROM, assessment of Quick-DASH and Mayo scores and radiographic documentation [9, 10].

To get an objective value of strength in the injured extremity anisometric strength analysis was done with a Dynamometer (Iso Force Control EV02, 10-400N, measurements in kg) in a 90 degree flexed elbow position and done in flexion and in extension in duplicate measurements to get reproducible results.

### Surgical technique

Patients were operated either in a prone or a lateral position. No tourniquet was used. The Triceps sparing approach was performed according to Bryan-Morrey [11, 12]. Articular fragments of the distal block were reduced under direct visualization and temporarily fixated with K-wires. Distal humerus plates (3.5 mm LCP) were placed dorso-laterally and medially in an orthogonal fashion. Extensor mechanism repair was done by reposition to its anatomic position along the posterior elbow and fixation of the bone chip to the olecranon with trans-osseous non-absorbable sutures.

## Results

Mean duration of follow-up was fifty-one months (range 24 months-12 years) in 24 patients, with an average age of 57.7 years (range 17-89 years). 13 were female, 11 male. According to the AO Classification there were five A2, one B1, two C1, nine C2 and seven C3 fractures.

### Range of motion

Mean flexion on the injured side was 138° (range 120-145), mean extension was 2° (range -5-15), mean pronation was 86° (range 70-90) and mean supination was 85° (range 65-90). The normal side revealed 139° of mean flexion (range 130-145), mean extension one degree (range -5-10), mean pronation 89° and mean supination 88° with ranges from 80-90°. The comparison of the injured and the not harmed side show no significant statistical differences (Student’s T-Test; flexion harmed versus normal p = 0.119, extension harmed versus normal p = 0.216, pronation harmed versus normal p = 0.11, supination harmed versus normal p = 0.135; significant difference defined as p < 0.05).

Mean arch of movement of 136 degrees was reached on the injured site.

### Strength

Strength was assessed by using the Iso-Cybex instrument at 90°. The mean strength of the injured side for extension was 8.46 kg (range 3-17.75 kg), the mean strength for flexion was 5.28 kg (range 2.1-10.8 kg). The mean extension strength of the normal side reached 9.15 kg (range 4.2-20.7 kg), the mean flexion strength was 5.25 kg (range 2.4-11.2 kg). There was no statistical difference in both directions comparing the harmed and the normal side. The Wilcoxon rank sum test with continuity correction revealed no difference between extension strength of normal side and injured side, p-value = 0.5092. The same holds true for the test for differences between normal and injured side regarding to flexion strength, p-value = 0.9835.

### Quick DASH score

The mean Quick DASH score was 10.3 (range, range 0-44 points). Pain assessment in Quick DASH had a mean value of 1.2 (range1-3). Work/regular activities scored with a mean value of 1.9 (range 1-4). One patient had a poor DASH result.

### Mayo elbow performance score

The mean Mayo score was 91 (range 75-100), indicating an excellent performance. Two patients had had a satisfying result.

### Radiographic assessment

Postoperative and follow up radiographs showed adequate fracture reduction and fracture healing in all patients. No step off more than one mm was seen. We measured correct extraarticular angles/axes of the distal humerus in both planes in all patients.

Radiologic follow up at least 22 months postop (mean 50 months, range 22-144 months postop) revealed in five cases a still visible osseous chip on the tip of the olecranon. Three patients had mild radiologic signs of posttraumatic arthritis (one patient with a previously diagnosed degenerative rheumatoid arthritic elbow joint) according to Jupiter’s scale [8].

### Complications

No wound infections were detected. Three patients had transient ulnar nerve palsies that had recovered completely at the last follow-up.

Three patients required reoperation. One for an early postoperative fixation failure of the humerus fracture. Another patient had a reoperation for an avulsion of the triceps tendon after a secondary trauma to the elbow, when he tried a handplant three months after the fracture fixation Hardware removal of the distal humeral plates and a reattachment/reconstruction of the triceps tendon avulsion was performed. The third patient’s hardware removal was combined with a neurolysis of the ulnar nerve with previous history of paraesthesia [13].

## Discussion

There is no consensus about the ideal surgical approach for ORIF of intraarticular distal humerus fractures. For many orthopedic surgeons the dorsal approach with a “Chevron”-osteotomy of the olecranon is still the standard approach. However, in recent years a variety of exposures are becoming used more frequently sparing the triceps muscle and tendon and preserving their continuity. Reecent results of triceps-preserving techniques have been shown by Habib et al., demonstrating an reasonable approach for C-fractures of the distal humerus by using an anocneus pedicle flip osteotomy. Potentinal functional impairment by irritating the intrinsic stability of the elbow has to be considered. Similar to our study a prospective randomised study and biomechanical evaltutions is needed for further judgement [8, 12, 14–16].

The main argument to use an olecranon osteotomy for ORIF of distal intraarticular humerus fractures is that it provides the widest exposure of the joint surface. Based on cadaveric studies different percentages of visualisation are reported. Wilkinson et al. demonstrated an exposed articular surface for the triceps splitting of 35%, for the triceps reflecting approach of 46% and for the olecranon osteotomy of 57%. “Chevron”-olecranon osteotomy exposure was not significantly better than the triceps reflecting approach [17]. Similar information is provided by Dakoure’et al. with 26, 37 and 52% [18].

However, an olecranon osteotomy has potential complications: failure to anatomically close the osteotomy at the end of the procedure, healing problems of the osteotomy (delayed union, non-union, secondary displacement), and hardware complications, many of them leading to secondary surgical procedures. Coles et al. identified 8% of elective removal of symptomatic osteotomy hardware [1]. Tak et al. showed that their osteotomies united in an average of 11 weeks (range, 8-20 weeks) with no non-unions but 4 delayed unions, which all healed by 20 weeks without any intervention [19]. Their most frequent complication were symptomatic osteotomy fixations in 19%, all of them needed removal of the implant after the osteotomy had united. 71% percent of the unsatisfactory results were seen in those patients who had symptomatic olecranon fixation. A study by Schmidt-Horlohe et al. reported on 31 patients with type-C-fractures of the distal humerus treated by ORIF via olecranon osteotomy and refixation of the osteotomy with hookplates. In this series removal of the hookplate was performed in 48.4% of patients [7, 20].

One of our senior authors (DR) has introduced the triceps reflecting approach described by Bryan and Morrey as an alternative to a “Chevron”-olecranon osteotomy for the surgical treatment of intraarticular distal humerus fractures in his practice in 2002. In this retrospective investigation of a consecutive clinical series of 24 patients we intended to answer two questions:

1. does the triceps reflecting approach offer sufficient exposure of the joint surface for anatomic reduction and stable internal fixation of the fracture; and,
2. does the triceps reflecting approach lead to functional impairment of the extensor apparatus as measured objectively.

Our results of the analysis of postoperative radiographs show an anatomic reconstruction of the joint surface and of the extraarticular angles in all cases, thus indicating that the exposure provided by the triceps sparing approach was adequate. Moreover, we observed that an intact olecranon can serve as a template for reconstruction of the trochlea, especially in osteoporotic bone with the risk of narrowing the trochlear width by compression due to poor bony resistance. Furthermore, closure saves time by reducing the reflected extensor apparatus back in place and fixing the the bony chip with a figure of eight non-resorbable transosseous suture.

The strength measurements revealed no statistically significant loss of objective function of the extensor apparatus on the injured side. In five patients, a slightly proximally displaced osseous chip was still visible on the last follow up X-ray, but this had no influence on the physician-rated strength measurements. Also, the subjective (patient-rated) scores (Quick DASH and Mayo Elbow Performance Score) showed only minimal impairment (10.3 and 91 pts. respectively). The ROM was uniformly satisfactory in all patients with no statistical differences to the non-injured side.

We therefore conclude that the triceps reflecting apporach to operatively treat distal intraarticular humerus fractures does not lead to functional disadvantages.

Bryan and Morrey have described the triceps reflecting approach in 1982 [8]. This exposure has been widely used predominantly for elbow arthroplasty. Although weakness in extension is commonly seen postoperatively, other complications such as infection, reoperation or loss of strength are rare. Guerroudj et al. did compare the in vitro mechanical properties of the triceps tendon after simulation of three common exposures and showed that all approaches resulted in a weakening of the triceps; however, the Bryan-Morrey lateral triceps-reflecting technique provided statistically better strength than V-Y or longitudinal splitting [11].

There are only few articles in the literature reporting on the use of a triceps sparing approach to the distal humerus in trauma. Ek et al. reviewed the functional outcome of seven complex distal humerus fractures managed with open reduction and internal fixation through a posterior triceps-sparing approach [21]. All their patients achieved good clinical scores. They postulate the posterior triceps-sparing approach to provide adequate exposure to the fracture site. Remia’s report on 9 adolescents showed an average triceps deficit compared with the uninvolved arm of 6-10% [22]. Compared with the Campbell triceps-splitting approach, no statistically significant difference in function or range of motion was found. They also propose the Bryan-Morrey triceps-sparing approach as a safe option for T-condylar distal humeral fractures in adolescents.

Regarding functional outcome and strength McKee et al. evaluated 25 isolated, closed, intra-articular distal humers fractures repaired operatively through a posterior approach (either olecranon osteotomy or triceps splitting). At follow-up (mean 37 months with arrange from 18 to 75 years) objective muscle-strength testing was performed. Significant decreases in mean muscle strength compared with that on the normal side were seen in both elbow flexion and elbow extension while no differences were shown between to two operative approaches. The mean DASH score of all patients was 20 points, indicating mild residual impairment. 12% of them had removal of prominent hardware used to fix the site of an olecranon osteotomy [23].

Our paper has a number of significant flaws to be considered. First, it is a retrospective study of a selected series of consecutive patients. The choice of the approach was made by personal preference of the surgeon. There is a considerable number of patients lost to follow up (7/31). Second, the number of patients that have been treated with an olecranon osteotomy at the two institutions during this same period of time is unknown. The paper therefore gives no information about what subset of patients might be preferably treated with an olecranon osteotomy to obtain a wider exposure of the joint in cases of more severe intraarticular comminution. However, it has been the observation of the authors that specially in patients with poor bone the exposure is sufficient. Third, the strength measurements have only been performed in 90° of flexion and not in other positions of the elbow. However, we believe that this measurement is representative enough to answer the question about objective loss of function regarding muscle strength.

## Conclusions

This investigation on 24 patients with distal intraarticular humerus fractures treated with ORIF using a triceps reflecting approach revealed excellent exposure regarding the quality of joint reconstruction and no relevant objective (strength) or subjective (Quick-DASH, Mayo Elbow Performance Score) functional impairment related to the surgical exposure. We conclude that the triceps reflecting approach for ORIF of distal intraarticular humerus fractures is a safe and valuable option. It avoids the potential complications of a “Chevron”-olecranon osteotomy.

## Abbreviations

AO: Association for the Study of Internal Fixation (Arbeitsgemeinschaft für Osteosynthesefragen)
DASH: Disabbillities of the arm, shoulder and hand
DR: Daniel Rikli
Et al.: Et alii
FU: Follow up
Kg: Kilogram
K- wires: Kirschner wires
LCP: Locking compression plate
LDI: Lukas Daniel Iselin
MJ: Marcel Jakob
ORIF: Open reduction internal fixation
Postop: Postoperative
RB: Reto Babst
ROM: Range of motion
TM: Tobias Mett
TRA: Triceps reflecting approach.
